# Clinical Spectrum of Postsurgical Parsonage-Turner Syndrome: A Perspective From an Electrodiagnostic Laboratory

**DOI:** 10.7759/cureus.41001

**Published:** 2023-06-26

**Authors:** Vasudeva G Iyer, Lisa B Shields, Yi Ping Zhang, Christopher B Shields

**Affiliations:** 1 Clinical Neurophysiology, Neurodiagnostic Center of Louisville, Louisville, USA; 2 Neurological Surgery, Norton Neuroscience Institute, Norton Healthcare, Louisville, USA

**Keywords:** neuralgic amyotrophy, neurological surgery, orthopedic surgery, electromyography, parsonage-turner syndrome, postsurgical, neurology

## Abstract

Background: Parsonage-Turner syndrome (PTS) is an underdiagnosed disorder characterized by the acute onset of severe pain in the shoulder/scapula/arm followed by muscle weakness/numbness in the distribution of nerves derived from the brachial plexus (BP). Surgical procedures are one of several antecedent events of PTS. This study describes the clinical spectrum of postsurgical Parsonage-Turner syndrome (PSPTS) in a large cohort of patients.

Materials and methods: Charts of patients diagnosed with PTS during a 16-year (2006-2022) retrospective review were analyzed to identify cases of PSPTS. The clinical criteria for PSPTS included the new onset of severe pain two days to four weeks after a surgical procedure followed by weakness of muscles innervated by one or more nerves arising from the BP. EDX criteria consist of denervation localized to branches of the BP. PSPTS cases were subdivided into two categories: definite PSPTS (surgery at a remote site) and probable PSPTS (surgery of the ipsilateral upper extremity or the cervical spine).

Results: Of 202 patients (204 episodes) diagnosed with PTS, 111 (54%) were idiopathic and 61 (30%) were PSPTS. Of the 61 PSPTS episodes, 26 were definite and 35 were probable PSPTS. The anterior interosseous nerve (AIN) was most affected, followed by the posterior interosseous (PIN), and suprascapular nerve.

Conclusion: In this series, surgery was the most commonly recognized antecedent event for PTS, and the AIN and PIN were the most frequent nerves affected. Surgeons should consider PTS in patients who develop postoperative severe shoulder pain and weakness of muscles innervated by the BP.

## Introduction

Parsonage-Turner syndrome (PTS), also known as neuralgic amyotrophy or acute brachial plexus neuropathy, is an underdiagnosed clinical entity [1]. Following Spillane’s report of “localized neuritis of the shoulder girdle” in 1943 [2], Parsonage and Turner described 136 cases of neuralgic amyotrophy, specifically, “the shoulder-girdle syndrome” [3]. The estimated annual incidence of PTS is 1.64 cases per 100,000 population [4,5], although a higher rate of 2-3/100,000 has also been reported [1,6]. Even the latter rate may be an underestimate [1]. Males are more commonly affected, and the highest incidence is in the 3rd-7th decades [4]. Right-sided symptoms are more frequent [7]. PTS is characterized by the acute onset of severe unilateral shoulder girdle/arm pain followed by progressive paresis and atrophy of the muscles innervated by one or more nerves derived from the brachial plexus [1,3,4,6-8]. The severe pain diminishes gradually, and although a dull ache may persist, often the muscle weakness tends to persist longer. Winging of the scapula, diminished pinch grip, and weakness of shoulder abduction, external rotation, and forearm pronation are frequently noted [1]. While any nerve of the brachial plexus may be affected, the upper and middle trunk distribution is the most common, specifically, the long thoracic, suprascapular, musculocutaneous, and/or axillary nerves [1,6,8,9]. The anterior interosseous nerve (AIN) and posterior interosseous nerve (PIN) have been reported to be more commonly involved in some series [6,10]. Diagnosis is based on the clinical picture and electrodiagnostic (EDX) studies; more recently magnetic resonance neurography (MRN) [11] and ultrasonography (US) have also been found to be valuable [12]. Unfamiliarity with the clinical features of this condition can potentially lead to misdiagnosis, unwarranted investigations, and unnecessary surgical intervention [4,13].

While no inciting factors are recognized in many patients, the known provoking events of PTS include viral illnesses (varicella zoster, herpes simplex, HIV, Coxsackie B virus, Hepatitis B, C, and E virus, Epstein-Barr virus, cytomegalovirus, SARS-CoV2), immunizations (tetanus toxoid/antitoxin, influenza, COVID-19, herpes zoster, typhoid, human papillomavirus (HPV), botulinum toxin), trauma, autoimmune disorders (systemic lupus erythematosus, polyarteritis nodosa, temporal arteritis), strenuous exercise, pregnancy/childbirth, certain medications (antiepileptics, antibiotics, immunosuppressants, antiretrovirals), and surgical procedures [1,3,4,6-8,12,14-16]. PTS may also have a hereditary origin [7,15,17].

Postsurgical PTS (PSPTS) is particularly important as it poses not only diagnostic and therapeutic challenges but also ushers in medicolegal issues. Several single cases and short series of PSPTS have been reported in the literature [5,7-9,13,15,18-25], however, a detailed analysis of a large series is not available. The goal of the present study is to provide better insight into the clinical spectrum of nerve involvement in PSPTS and the type of preceding surgical procedures. The unique features of PSPTS are highlighted, and the mechanism of this condition is discussed.

## Materials and methods

Under an Institutional Review Board (IRB)-approved protocol, we performed a 16-year (2006-2022) retrospective analysis of patients referred for EDX studies who were diagnosed with PTS. The EDX studies were performed in our American Association of Neuromuscular and Electrodiagnostic Medicine (AANEM)-accredited facility using a standard protocol of our laboratory [26]. The inclusion criteria for the diagnosis of PSPTS included the acute onset of pain in the shoulder girdle/upper extremity followed by weakness in the distribution of one or more nerves arising from the brachial plexus and confirmed by denervation changes in the affected muscles by needle electromyography (EMG). To be considered PSPTS, a surgical procedure must have preceded the onset of symptoms within a window of 48 hours to four weeks. Thus, patients in whom positional pressure or perioperative injury to the nerves was more likely were excluded. Patients in whom the surgical procedure was anatomically remote from the brachial plexus were considered definite PSPTS. Those in whom the surgery was in the vicinity of the brachial plexus (cervical spine and shoulder) or in the ipsilateral upper extremity were classified as probable PSPTS. Several metrics were collected including gender, age, side of symptoms, location of pain, affected nerves, type of surgery, and duration between surgery and symptom onset.

Informed consent was obtained from all patients. The University of Louisville IRB determined that our study was exempt according to 45 CFR 46.101(b) under Category 4. The IRB number is 22.0909.

## Results

Clinical findings and type of surgery

A total of 202 patients were diagnosed with PTS. The total number of PTS episodes was 204 (one patient had three episodes). The PTS was idiopathic in 111 (54%) episodes; among those in whom an antecedent event could be identified, the most common group was postsurgical (61 (30%)), followed by post-infection/vaccines (24 (12%)), heavy exertion/trauma (6 (3%)), and miscellaneous medical procedures groups (acupuncture, colonoscopy) (2 (1%)).

The mean age of the 61 patients with PSPTS was 56 years (range: 19-86 years), and the majority (42 (69%)) of patients were male (Tables 1, 2).

**Table 1:** Definite Postsurgical Parsonage-Turner Syndrome Table 1 includes only reports where the brachial plexus or one or more of the nerves derived from the brachial plexus are affected and the involved nerve or nerves are precisely identified. AIN: anterior interosseous nerve PIN: posterior interosseous nerve Key: Location of Pain UE: Whole upper extremity N: Neck Sh: Shoulder Sc: Scapular E: Elbow A: Upper arm F: Forearm H: Hand

Patient #	Gender (M/F)	Age (Years)	Side (L/R)	Location of Pain	Affected Nerves	Type of Surgery
1	F	51	R	Sh	Axillary	Cholecystectomy
2	M	52	R	E	PIN	Abdominal surgery
3	F	45	R	F	AIN	Pelvic surgery
4	M	30	L	Sc, Sh	Suprascap	Hernia repair
5	M	86	R	Sh	PIN	L thoracotomy
6	M	66	R	Sh	Musculocut, Median	Aortic aneurysm repair
7	M	37	L	Sc	Suprascap	Cholecystectomy
8	M	60	L, R	Sc, A	Radial, Median	Abdominal surgery
9	F	44	R	F	AIN	Hysterectomy
10	M	63	R	A	Lower trunk	Cholecystectomy
11	M	32	R	F	AIN	Hernia repair
12	F	34	R	Sc, UE	Axillary, Suprascap	L wrist surgery
13	M	71	R	Sc	AIN	Hip surgery
14	M	56	R	Sh, Sc	Suprascap	L hand ganglion cyst excision
15	F	72	L	Sc, E	PIN	Removal of breast implant
16	F	62	L	F	AIN	Reattachment of Achilles tendon
17	M	62	R	UE	PIN	Thyroid surgery
18	M	62	R	UE	Median	Knee surgery
19	F	60	L	UE	Lower trunk	Cholecystectomy
20	M	74	R	UE	PIN	Amputation of 4^th^ toe
21	F	64	R	E	Ulnar, PIN	L shoulder surgery
22	M	19	L	Sc, Sh	Suprascap	Hip surgery
23	M	49	R	Sh	Axillary N sensory	Cholecystectomy
24	F	69	R	Sh	Axillary	Knee surgery
25	M	33	L	Sh, A	Suprascap	Foot bunionectomy
26	F	63	L	Sh	PIN	Abdominal surgery

**Table 2:** Probable Postsurgical Parsonage-Turner Syndrome AIN: anterior interosseous nerve PIN: posterior interosseous nerve KEY: Location of Pain UE: whole upper extremity N: neck Sh: shoulder Sc: scapula A: upper arm F: forearm H: hand E: elbow

Patient #	Gender (M/F)	Age (Years)	Side (L/R)	Location of Pain	Affected Nerves	Type of Surgery
1	M	59	L	Sh	AIN	Coronary artery bypass
2	M	56	R	UE	AIN	R finger repair
3	M	47	R	A	Median	R palm surgery
4	M	65	L	E	Upper trunk	Cervical spine surgery
5	M	65	R	Sc	PIN	Cervical spine surgery
6	M	31	R	Sh	Long thoracic	R wrist surgery
7	M	57	R	F	Median	R shoulder surgery
8	M	38	L	Sc, Sh	Musculocut	L cubital tunnel release
9	M	47	L	E	AIN	L shoulder surgery
10	M	53	R	Sc	Lower trunk	R shoulder surgery
11	M	51	R	Sh	AIN	R shoulder surgery
12	F	60	R	Sh	AIN	R shoulder surgery
13	M	74	R	Sh, UE	Axillary, lower trunk	R shoulder surgery
14	M	74	L	F	PIN, Median, Ulnar	Coronary artery bypass
15	F	54	R	N, Sh	Median, AIN	Cervical spine surgery
16	F	61	R	Sh	Upper trunk	Cervical spine surgery
17	M	54	R	A	Post cord	Cervical spine surgery
18	M	57	R	F	AIN	Elbow repair biceps tendon reattachment
19	F	60	R	Sh, A	Lower trunk	R shoulder surgery
20	F	56	L	E	Upper trunk	Repair fracture distal L humerus
21	F	67	R	UE	Lat cord, Median	R elbow repair
22	M	44	R	Sh	Suprascapular	R shoulder surgery
23	M	44	R	A, Sh	Long thoracic	R shoulder surgery
24	M	58	L	F	AIN	L shoulder surgery
25	M	70	L	UE	Median	L shoulder surgery
26	F	58	R	F	AIN	R shoulder surgery
27	M	55	L	UE	AIN	Cervical spine surgery
28	M	44	L	F	AIN	L ulnar shortening
29	F	57	L	UE	Median	L shoulder surgery
30	F	69	R	UE	AIN	R shoulder surgery
31	M	60	L	E	Median, AIN	L shoulder surgery
32	M	73	L	US	Lower trunk	Coronary artery bypass
33	M	63	R	Sc, Sh, N	AIN	Cervical spine surgery
34	M	66	R	H	AIN	R shoulder surgery
35	M	58	R	No pain	AIN	R shoulder surgery

Table 1 includes only reports where the brachial plexus or one or more of the nerves derived from the brachial plexus are affected and the involved nerve or nerves are precisely identified. The PSPTS occurred on the right side in 39 (64%) patients, left in 21 (34%), and bilateral in one (2%). The mean duration between the surgery and symptom onset was two weeks (range: three days to four weeks). The shoulder was the most frequent location of pain in 22 (36%) episodes, followed by the entire upper extremity in 13 (21%), the scapula in 12 (20%), and the forearm in 10 (16%).

The type of inciting surgical procedure varied with no well-defined pattern. The most common type of surgery was of the shoulder and elbow/hand in 18 (30%) and 10 (16%) episodes, respectively (Table 3).

**Table 3:** Types of Surgery in Postsurgical Parsonage-Turner Syndrome PSPTS: postsurgical Parsonage-Turner syndrome

Types of Surgery	Total Numbers of PSPTS Episodes (n=61)	Number of Definite PSPTS Episodes (n=26)	Number of Probable PSPTS Episodes (n=35)
Shoulder	18 (30%)	1	17
Elbow/hand	10 (16%)	2	8
Cervical spine	7 (11%)	0	7
Cholecystectomy	5 (8%)	5	0
Other abdominal	4 (7%)	4	0
Hip and knee	4 (7%)	4	0
Coronary artery bypass	3 (5%)	0	3
Foot	3 (5%)	3	0
Pelvic	2 (3%)	2	0
Hernia repair	2 (3%)	2	0
Thoracotomy	1 (2%)	1	0
Breast	1 (2%)	1	0
Thyroid	1 (2%)	1	0

Ipsilateral upper extremity surgery preceded 25 (41%) episodes, and seven (11%) episodes occurred following cervical spine surgeries. Three patients experienced PSPTS following coronary artery bypass surgery. Coronary artery bypass surgery involved sternal splitting and retraction which may cause traction injury to the brachial plexus. In such cases the distinction between PSPTS and brachial plexus injury is difficult and, hence, these cases are considered probable PSPTS.

Electrodiagnostic studies

Needle EMG showed evidence of denervation in the form of fibrillations and positive waves. Muscles innervated by a single nerve such as the AIN or suprascapular nerve usually showed maximum denervation, but lesser degrees of denervation changes were not uncommon in additional muscles, suggesting trunk or cord involvement of the brachial plexus.

Of the 61 patients who experienced PSPTS, a single nerve was involved in 52 (85%) patients, while nine (15%) involved multiple nerves (eight episodes with two nerves and one episode with three nerves) (Table 4).

**Table 4:** Affected Nerves in Postsurgical Parsonage-Turner Syndrome PSPTS: postsurgical Parsonage-Turner syndrome AIN: anterior interosseous nerve PIN: posterior interosseous nerve BP: brachial plexus * Multiple nerves: Axillary/Suprascapular: 1, Musculocutaneous/Median: 1, Radial/Median, Ulnar/PIN: 1 ** Multiple nerves: Median/AIN: 2, PIN/Median/Ulnar: 1, Axillary/Lower trunk: 1, Lateral Cord/Median: 1

Nerve	Total Numbers of PSPTS Episodes (n=61)	Number of Definite PSPTS Episodes (n=26)	Number of Probable PSPTS Episodes (n=35)
AIN	19 (31%)	5	14
PIN	7 (11%)	6	1
Suprascapular	6 (10%)	5	1
Median	5 (8%)	1	4
Upper trunk	5 (8%)	2	3
Lower trunk	3 (5%)	0	3
Axillary	3 (5%)	3	0
Long thoracic	2 (3%)	0	2
Musculocutaneous	1 (2%)	0	1
Posterior cord of BP	1 (2%)	0	1
Lateral cord of BP	0 (0%)	0	0
Multiple nerves	9 (15%)	4 *	5 **

The AIN was most often involved (19 (31%) patients), followed by the PIN (seven (11%)) and the suprascapular nerve (six (10%)).

Most patients underwent the initial EDX studies within 3-4 months after symptom onset. Reinnervation changes in the form of polyphasic motor units were often seen at that time. Patients with isolated AIN or PIN involvement often showed poor reinnervation. The opportunity for serial EMG studies was available in only six patients, and partial reinnervation occurred in these cases after six months following the first EDX study.

Illustrative cases

Case #1: Definite PSPTS

**Figure 1:**
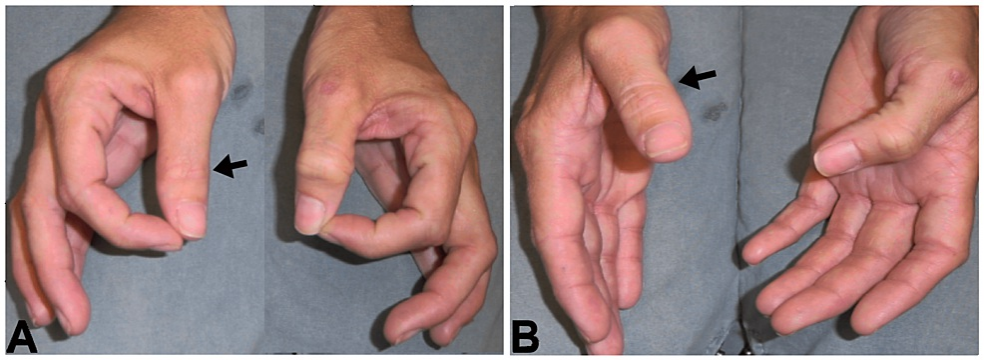
Definite Postsurgical Parsonage-Turner Syndrome with Anterior Interosseous Nerve Palsy Patient with definite postsurgical Parsonage-Turner syndrome with right anterior interosseous nerve palsy. (A) Positive ‘O’ sign. (B) Weakness of the flexor pollicis longus. Arrows indicate an inability to flex the flexor pollicis longus.

A 62-year-old female underwent repair of a torn Achilles tendon. Two weeks later she woke up with severe pain in the right shoulder and forearm. One day later the patient experienced weakness in the right hand. The pain subsided after two weeks, but the weakness persisted. Neurological examination revealed the weakness of flexion of the thumb and index finger at the interphalangeal joints (Figure 1A, 1B).

EDX studies three weeks later showed denervation of the flexor pollicis longus (FPL) and pronator quadratus (PQ) confirming AIN palsy. This is considered an example of definite PSPTS due to the occurrence of PTS following surgery at a remote anatomical site.

Case #2: Probable PSTS

**Figure 2:**
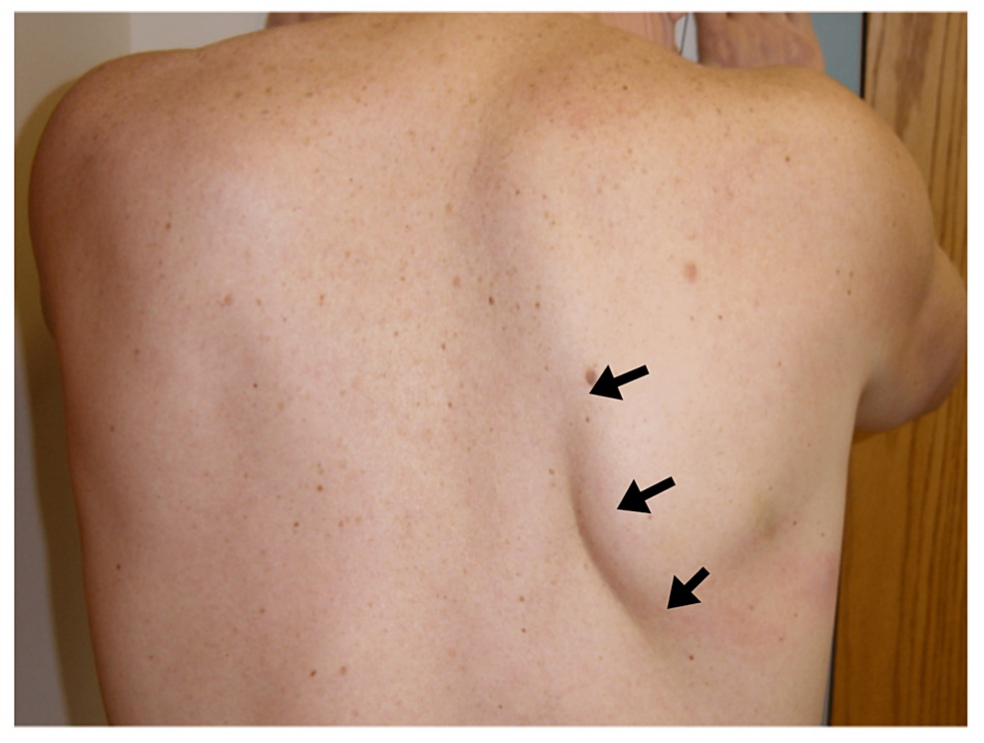
Probable Parsonage-Turner Syndrome with Long Thoracic Nerve Palsy Patient with probable Parsonage-Turner syndrome with right long thoracic nerve palsy causing winging of the scapula (arrows).

A 31-year-old male developed right proximal upper extremity weakness after undergoing a surgical procedure in the right wrist. Severe pain in the right scapular area was noted after four days, which persisted for three weeks. Weakness of right shoulder movements was noted two days after the onset of scapular pain. Examination showed winging of the right scapula from serratus anterior muscle weakness supplied by the long thoracic nerve (Figure 2).

Needle EMG confirmed the presence of denervation changes in that muscle. There has been only partial improvement at six months. This case is grouped under probable PSPTS as the surgery was in the ipsilateral upper extremity.

Case #3: Recurrent PSPTS

**Figure 3:**
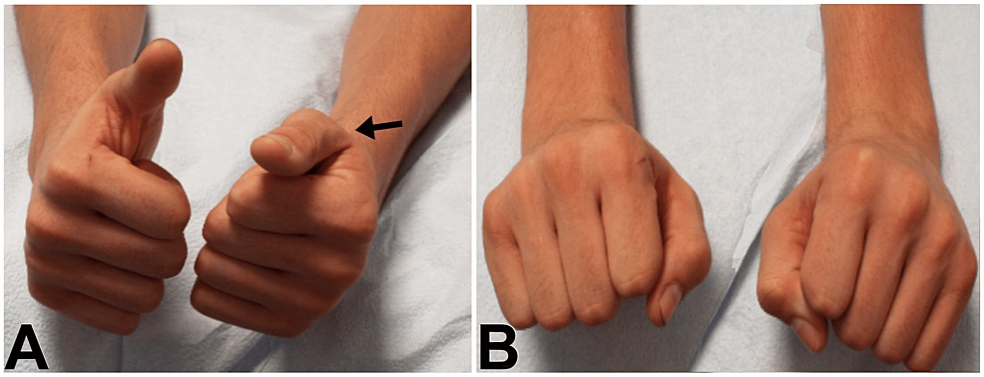
Recurrent Episodes of Postsurgical Parsonage-Turner Syndrome Patient with recurrent episodes of postsurgical Parsonage-Turner syndrome. (A) Unable to perform a “thumb up” sign on the left (arrow). (B) Normal dorsiflexion of the wrist. Finger drop without wrist drop is diagnostic of posterior interosseous nerve neuropathy. Finger drop with wrist drop is radial nerve neuropathy.

A 52-year-old male reported severe pain and weakness in the right upper extremity two weeks after a right knee replacement. Neurological examination revealed weakness of the right median nerve-innervated muscles, most prominently the FPL. EDX studies showed denervation changes in the FPL and to a lesser extent in the pronator teres (PT), flexor carpi radialis (FCR), and abductor pollicis brevis (APB). His symptoms gradually improved over 3-4 months. Six months following the knee surgery, the patient underwent the removal of a benign thyroid nodule. Three weeks later he noted pain and weakness in the left upper extremity with finger drop and an inability to extend the left thumb (Figure 3A, 3B).

EDX studies four weeks later confirmed denervation in the left PIN distribution. He regained muscle strength gradually within 10-12 months. Four years following the thyroid surgery, the patient underwent a left rotator cuff repair and biceps tenotomy. Once again, he suffered intense pain in the left upper extremity and weakness of the left hand. Neurological examination revealed weakness in the left AIN distribution. EDX studies four weeks later showed denervation of the FPL and PQ. The symptoms resolved within six months. The first two episodes are examples of definite PSPTS, but the third episode was classified as probable PSPTS as the surgery involved the ipsilateral upper extremity.

## Discussion

PSPTS should be considered in the differential diagnosis of a postsurgical patient with severe shoulder pain and weakness in the distribution of one or more nerves derived from the brachial plexus. The diagnosis of PSPTS occurring after surgery at an anatomically remote site is obvious; however, when antecedent surgery is in the ipsilateral upper extremity or cervical spine, perioperative injury to the brachial plexus or cervical nerve roots must be considered in the differential diagnosis. When a patient experiences PTS following cervical spine surgery, it is important to differentiate between a potential complication of the cervical surgery (such as C5 palsy) from PSPTS [13]. Approximately 5% of patients undergoing cervical decompression are complicated by postoperative weakness [18]. Furthermore, the incidence of C5 palsy reportedly varies between 1% and 29% [27]. Diagnostic features that may signify PSPTS are (1) a 48-hour to one-week delay between surgery and symptom onset (not immediately postoperatively); (2) intolerable pain disproportionate to the expected perioperative course followed by the onset of muscle weakness with gradual improvement of pain; (3) non-congruent distribution of pain, sensory abnormalities, and weakness; and (4) confirmation of specific nerve denervation on EDX studies [9,13].

EDX study is the most important modality after neurological examination in diagnosing PSPTS. An EMG is able to identify, localize, and grade the severity of denervation and re-innervation of the affected muscles [4,9]. In PSPTS there is often widespread denervation of the involved muscles characterized by fibrillation potentials and positive waves which appear approximately 2-3 weeks postoperatively [4,6,7]. Denervation changes are usually more prominent in muscles supplied by a single nerve. Serial EMGs may detect muscle re-innervation, exemplified by high amplitude wide duration polyphasic motor unit potentials approximately three months after surgery [5,6]. Nerve conduction velocity studies often demonstrate low amplitude/absent compound muscle action potentials (CMAP) and/or sensory nerve action potentials (SNAP) indicating axonal degeneration in the brachial plexus and/or in multiple nerves originating from the plexus [5]. Although the EDX study is the gold standard for diagnosing PSPTS, an MRI of the cervical spine or brachial plexus may be performed to rule out other neurologic or musculoskeletal pathologies such as nerve root compression or a mass lesion [4]. MR imaging may also reveal an inflammatory response in the brachial plexus in the acute phase [11]. Hourglass-like fascicular constrictions, torsions, and entwinement have been described in the affected nerves in US studies [28,29].

The pathophysiology of PSPTS is speculative. Proposed mechanisms include (1) the stress of surgery may activate an unidentified virus lying dormant in the nerve roots [8,15] or (2) surgery may trigger a systemic allergic hypersensitivity or immune-mediated inflammatory process targeting the peripheral nerve associated with PSPTS [8,9,13,15]. This condition is precipitated by surgical intervention but is probably unrelated to technical, mechanical, structural, compressive, or traumatic events such as traction injury of the brachial plexus or pressure palsies secondary to positioning [7,9,18]. This is exemplified by the definite form of PSPTS, which occurs after surgery at a remote anatomical site. The nerve biopsy in cases of post-surgical inflammatory neuropathy, the mononeuropathy form of which resembles PSPTS, showed increased epineural perivascular lymphocytic inflammation and increased axonal degeneration; these authors believe that immunotherapy may have led to improvement [9].

Myriad single cases and short series of PSPTS have been reported in the literature spanning a wide array of surgical procedures: orthopedic (knee arthrotomy/replacement, cervical spine decompression, hip replacement), gynecologic (vaginal hysterectomy, hysteroscopy, pubovaginal sling), and abdominal (appendectomy) (Table 5) [5,7-9,13,15,18-25].

**Table 5:** Postsurgical Parsonage-Turner Syndrome in the Literature D&C: dilation and curettage L: left R: right B: bilateral AIN: anterior interosseous nerve PIN: posterior interosseous nerve CTR: carpal tunnel release ACDF: anterior cervical discectomy and fusion

	Study	Number of Patients	Affected Nerve	Type of Surgery
1	Malamut et al. [22]	6	R long thoracic	Tonsillectomy
			R long thoracic, suprascapular	Appendectomy
			L post interosseous, suprascapular, lower trunk	1^st^ rib resection
			L long thoracic, R long thoracic, R suprascapular	Vaginal hysterectomy
			L long thoracic, L suprascapular, L lower trunk, L thoracodorsal, R long thoracic, R suprascapular, R lower trunk	Coronary artery bypass
			L AIN, L PIN	Knee arthrotomy
2	Eggers and Asai [7]	1	Lower trunk	Knee replacement
3	Fibuch et al. [8]	1	Upper trunk	Hysteroscopy, D&C
4	Griffin et al. [5]	1	R axillary	Mastectomy for gynecomastia
5	Horlocker et al. [20]	1 (2 episodes)	Upper > lower brachial plexus	B shoulder surgery
6	Szwejbka et al. [25]	1	B AIN	Abdominal surgery
7	Huang et al. [21]	1	Lower trunk	Orthognathic surgery
8	Sisco and Dumanian [23]	3	AIN	Shoulder surgery
9	Squintani et al. [24]	1	B AIN	R CTR
10	Shetty et al. [15]	1	L AIN	Total hip replacement
11	Verhasselt et al. [13]	1	Axillary, musculocutaneous	ACDF C5-7
12	Barrington et al. [9]	1	Upper trunk	Reconstruction thumb from toe
13	Butt et al. [19]	1	Suprascapular, axillary	Dental surgery

Squintani and colleagues report a 66-year-old man who underwent a left carpal tunnel release and experienced aching of the wrist and bilateral weakness of the thumb and index fingers several days later [24]. EDX tests revealed bilateral AIN neuropathy, and serology confirmed an anti-nuclear antibody positivity. The patient had been diagnosed with right PTS several months earlier. These authors suggest immunological pathogenesis with the relapses of bilateral acute AIN neuropathy [24]. Horlocker and colleagues reported a diabetic patient who experienced PSPTS four months apart after undergoing two shoulder procedures [20]. The unusual case described in the present study with three separate PSPTS events resembles these previously reported cases and further corroborates the immune-mediated inflammatory mechanism involved in PSPTS.

Of the 136 patients in Parsonage and Turner’s study, 98 (72%) reported a precipitating factor [3]. Of the 98 patients with a precipitating factor, infection was the most common factor (71 (72%) cases) followed by surgery (12 (12%) cases, with hernia repair in eight cases). In van Alfen and colleagues’ study of 246 cases of PTS, 199 were idiopathic PTS, while 47 were hereditary [17]. Based on the clinical history, 53.2% of patients reported an antecedent event, with infection as the most common (43.5%) followed by exercise (17.4%) and surgery (13.9%). Most patients experienced motor paresis in the upper and/or middle plexus including the long thoracic nerve. Only nine patients had a predominance of the AIN. In Ferrante and Wilbourn’s study of 281 patients (322 bouts of PTS, 57 of which were bilateral, for a total of 379 events) with sporadic PTS who underwent EDX testing, a single nerve was involved in 174 events (46%), and 205 (54%) were multifocal [30]. Pure motor nerve involvement was most commonly observed and greatly surpassed that of mixed sensorimotor nerves. These authors suggested that distal motor branch involvement accounted for the severe single muscle wasting and weakness that frequently accompany PTS [30]. In our previous study of 38 cases of PTS over nine years, an inciting event was reported in 16 (42%) cases; 14 (87%) of these cases followed a surgical procedure [6]. The most frequently involved nerves were the AIN and PIN, comprising 16 (42%) cases.

Among the patients with PTS seen in our current study, an antecedent surgical procedure was the most reported antecedent event. In cases of PTS after surgical procedures on the ipsilateral upper extremity, it is often difficult to rule out perioperative nerve injury. Thus, we included these cases as probable PSPTS in which the symptoms started more than two days following surgery. Occasionally, this distinction can be challenging, especially with an incomplete history or when medicolegal issues are involved. In cases where the surgical procedure is at a remote site, and a single nerve palsy such as AIN, PIN, long thoracic nerve, or suprascapular nerve exists, definite PSPTS is more likely to occur. Finger drop without wrist drop is diagnostic of PIN neuropathy (Figure 3B). Finger drop with wrist drop is radial nerve neuropathy.

There are no specific features that can predict the occurrence of PSPTS. Patients with hereditary PTS have a genetic abnormality that makes them vulnerable to recurrent episodes of PTS. However, this presentation may occur without specific factors and does not confirm the diagnosis of PSPTS. The antecedent surgery does not predict the topography of nerve involvement. The AIN and PIN were the most common single nerves affected in our study which differs from other series [17]. This finding may reflect referral bias as patients with hand weakness are referred from the Hand Clinic.

Prompt recognition of the signs and symptoms associated with PSPTS may avoid unnecessary medical testing and/or surgery [4,13]. Recognition in the acute phase is important as various treatment options including high-dose dexamethasone, intravenous immunoglobulin, opioid medications for neuropathic pain, non-steroidal anti-inflammatory medications (NSAIDS), carbamazepine, gabapentin, amitriptyline, and physical therapy can be initiated earlier [1,4,6,13]. Most cases of PTS are not diagnosed at the initial visit, and it often takes evaluation by a physician familiar with PTS and an EMG study. By then the window of opportunity to treat with corticosteroids may be over. PTS is a self-limiting condition, usually lasting between one and two weeks in duration although pain and paresis may persist for several years [1,4 17]. The total return of muscle strength in PTS is rare, especially in patients with AIN or PIN palsy. Conduction block at the level of the brachial plexus has been reported [6], but almost all cases in this series showed denervation changes indicating axonal injury, which has a less favorable prognosis than conduction block. For patients with intractable symptoms, surgery consisting of neurolysis, nerve resection with interposition grafting, or tendon transfer may be warranted [6].

Strengths and limitations of the current study

The strengths of the current study include the large number of patients who were diagnosed with either definite or probable PSPTS following varied types of surgical interventions, all of whom underwent EDX studies. The limitations of the study include its retrospective nature and lack of follow-up in the majority of patients after the EDX studies. Therefore, we were unable to document their treatment course or ascertain whether they attained improvement in their symptoms. The lack of MR imaging and US studies of the brachial plexus and its branches is another limitation. An additional limitation is that cervical MRIs were not performed after the onset of new symptoms in the postoperative period in patients who underwent cervical spine surgery and subsequently developed PSPTS. The pattern of clinical and EMG findings was not supportive of radiculopathy in these patients and suggested the involvement of the brachial plexus or one or more of its branches (AIN, PIN).

## Conclusions

In our current study, surgery was the most commonly recognized antecedent event for PTS. Furthermore, the AIN was most commonly affected, followed by the PIN and the suprascapular nerve. Surgeons should have a high index of suspicion of PSPTS in patients with severe shoulder pain shortly after surgery followed by significant weakness of muscles innervated by the brachial plexus. EDX, MRI, and high-resolution US studies are valuable in differentiating PSPTS from other conditions that share a similar semiology.
